# Mixed Metal Allergy Ancillary to Surgical Staples

**DOI:** 10.7759/cureus.26899

**Published:** 2022-07-15

**Authors:** Naveed E Ahmed, Rio Varghese, Ameen Abdel-Khalek, Ammarah Tariq, Tony Yu, Sakthi Ganeshalingam, Chantae C Hollis, Elizabeth O Amos-Arowoshegbe, Kalsuum Nasser Deen, Frederick Tiesenga

**Affiliations:** 1 Medicine, Saint James School of Medicine, Park Ridge, USA; 2 Medicine, Saint James School of Medicine, West Virginia, USA; 3 Medical Student, Saint James School of Medicine, The Quarter, AIA; 4 Surgery, Richmond Gabriel University, Kingstown, VCT; 5 Psychiatry and Behavioral Sciences, Bermuda Hospitals Board, Paget, BMU; 6 Psychiatry, John F. Kennedy University School of Medicine, Willemstad, CUW; 7 Medicine, Windsor University School of Medicine, Basseterre, KNA; 8 Internal Medicine, Washington University of Health and Science, San Pedro, BLZ; 9 General Surgery, West Suburban Medical Center, Chicago, USA

**Keywords:** allergen-producing metal composites, hypersensitivity reactions, acute allergic reaction, intra-operative cholangiogram, conventional laparoscopic cholecystectomy, titanium allergy, zirconium allergy, nickel allergy, surgical staples, metal allergies

## Abstract

Metal allergies to surgical clips are atypical and often neglected as a differential. It becomes even more unexpected when there is a lack of relevant medical history of the patient reacting poorly to known metals. This study entails the unique finding of newly discovered hypersensitivity to nickel and zirconium, metals commonly found in surgical clips, in a 28-year-old female who underwent laparoscopic cholecystectomy with a cholangiogram.

## Introduction

Metal allergies are a concern in the surgical field due to the possibility of patients developing hypersensitivity reactions [1]. Surgical devices are ordinarily composed of titanium, cobalt, zirconium, and nickel [1]. Nickel is one of the most common allergens visited in literature [2,3], with reports of nearly 20% of North American patients receiving a positive test patch [2], and especially common in women [3]. Hypersensitivity reactions to metal composites can result in contact dermatitis, pustulosis palmoplantar, lichen planus, dyshidrotic eczema, or burning mouth syndrome [4]. However, post-operatively allergic reactions may also present with non-specific symptoms such as pain, swelling, itching, warmth, or failure of the device [5], making diagnosing and treating hypersensitivity reactions a challenge.

Laparoscopic cholecystectomy has become the gold-standard treatment for common gallbladder diseases, such as cholecystitis, cholelithiasis, and biliary dyskinesia, to name a few. Recently, studies have shown its benefit in the symptomatic relief of biliary pain due to disorders such as biliary hyperkinesia [6-8]. Surgical clips are used in laparoscopic cholecystectomy for cystic duct and artery control [9]. Like most surgical instruments, they contain allergen-producing metal composites.

## Case presentation

A 28-year-old female successfully underwent laparoscopic cholecystectomy with a cholangiogram with surgical clips. Prior to the surgery, the patient had been complaining of right upper quadrant pain as well as nausea. A paraisopropyl-iminodiacetic acid (PIPIDA) scan showed a gallbladder ejection fraction of 95%, so with a tentative diagnosis of biliary hyperkinesia, laparoscopic cholecystectomy was performed. This surgery was uncomplicated with no bile leak or spillage and no notable intra-operative finding.

The patient reported that she developed fatigue, cognitive impairment, slowed thought processes, tinnitus, dizziness, light and sound sensitivity, and nasal congestion roughly three days after the surgery. Swelling and soreness on her right flank and erythema developed two weeks later. Preliminary differentials were pancreatitis, acute sclerosing cholangitis, nephrolithiasis, decompensated liver cirrhosis, and endocrine-associated anomalies such as pituitary or abnormal thyroid function. Series of imaging such as ultrasound, X-ray, CT, and MRI, along with blood tests such as CBC, CMP, BMP, infectious panel, TSH, and coombs tests, all ruled out possible considered differentials. ESR and mild leukocytosis were found in blood work.

**Figure 1:**
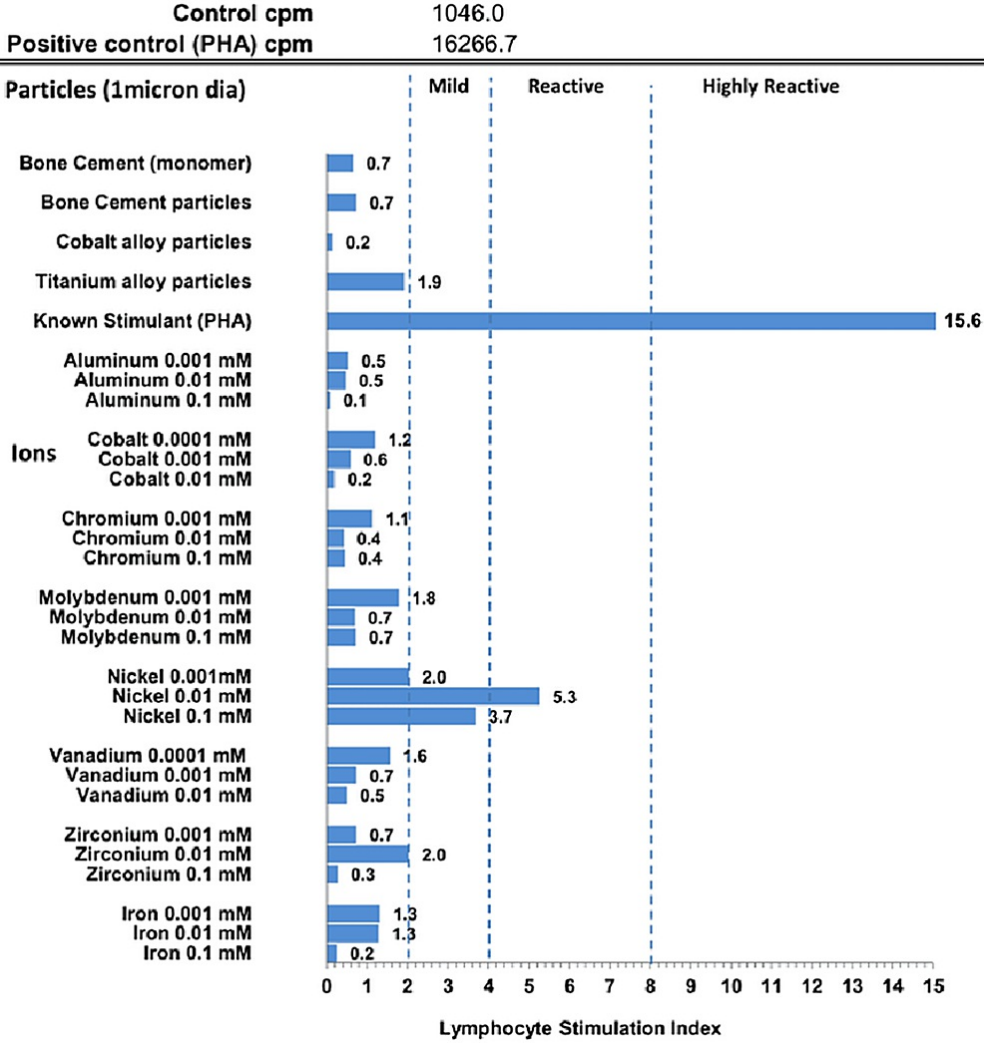
Metal lymphocyte transformation test (LTT) shows reactivity to nickel and zirconium with titanium alloys almost at the threshold cpm, counts per minute; PHA, Phytohemagglutinin; LTT, Lymphocyte transformation test; dia, Diameter

Metal allergy was considered, and a skin patch test and lymphocyte transformation test (LTT) was ordered, which showed reactivity for nickel and zirconium and sensitivity toward titanium approaching threshold values (Figure 1). Surgical clips used in this patient during the laparoscopic cholecystectomy were the culprit since most common surgical clips are derived from an alloy containing primarily titanium, aluminum, vanadium, and nickel. The risk of injury with removing the surgical clips to proximal structures such as the common bile duct and the proper hepatic artery was substantial, so the patient deferred to be treated medically and was discharged.

**Figure 2:**
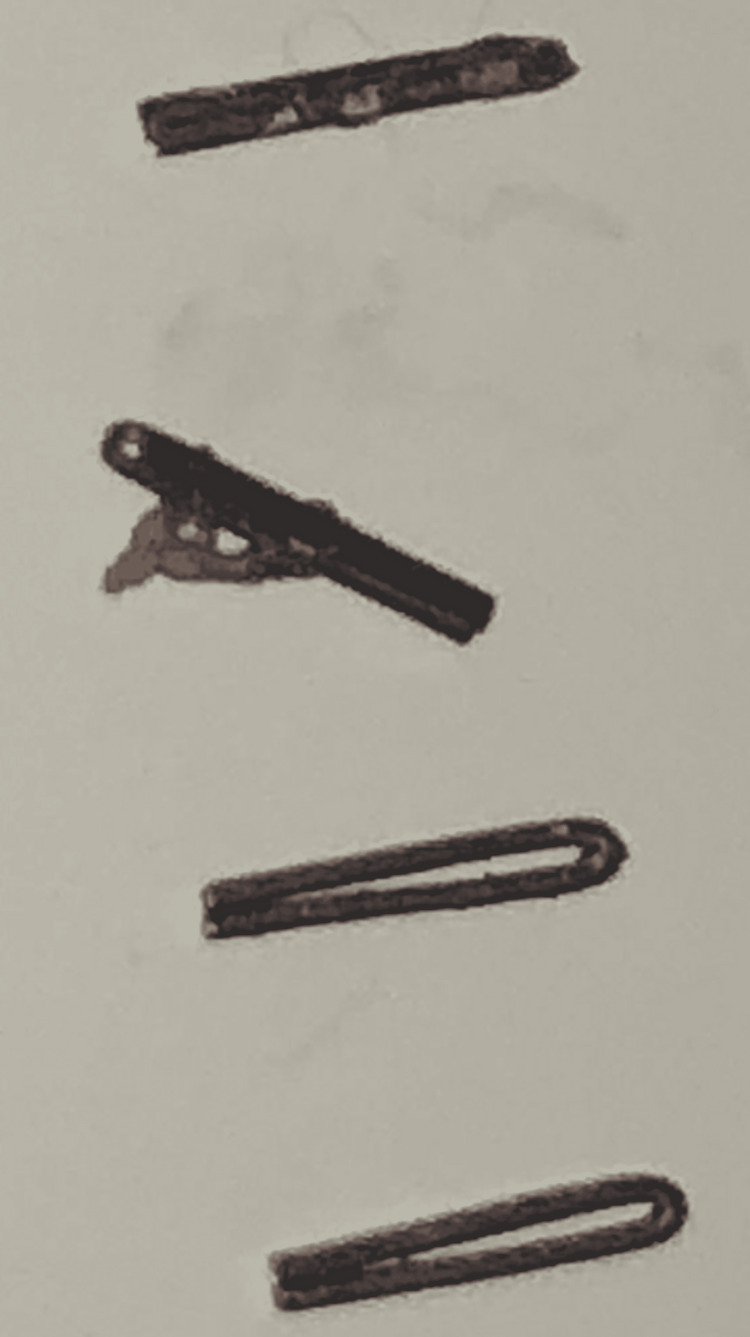
Four surgical clips were removed from the site of the previous laparoscopic cholecystectomy

Four months after the laparoscopic cholecystectomy, the patient returned to the outpatient clinic for a follow-up with her surgeon due to increased flank pain with sensitivity to touch. She defined the pain as a sharp stabbing, localized to the right upper quadrant. The patient also developed dry and sensitive skin, occasional itching, tingling in the palms of her hands, and soreness in her hands. The patient agreed to the second surgery to remove surgical clips as her symptoms did not improve with different diets or activities. The surgery for removing the clips took place roughly within a month of this visit, and it was uneventful, with no notable intraoperative findings (Figure 2).

The patient conveyed improvement in symptoms almost immediately after the surgery. Within days of surgery, nasal congestion and numbness/tingling in her arms had dissipated. There was a notable reduction in the intensity of pain and other symptoms. The resolution of symptoms post removal of staples lent significant credence to the preliminary diagnosis of metal allergy.

## Discussion

The use of surgical clips is highly standard in many surgeries. In the case of cholecystectomy, they are used to prevent the leakage of bile acids, amongst other reasons. However, as this case demonstrates, allergy testing is highlighted and should be a common practice pre-operatively to prevent any easily preventable postoperative adverse events. Most allergy tests are relatively inexpensive and are covered by most insurance plans as long as an indication is provided. The cost of a repeat surgery is much higher relatively. Nonetheless, more research is required to confirm the cost-effectiveness of regular allergy testing during the pre-operative window.

For this patient, a metal allergy test was not done before surgery due to no prior history of metal allergies. The patient, in this case, was found to be reactive to nickel and zirconium, fundamental components of many surgical clips/staples. Presensitization to a significant amount of nickel is sufficient to trigger an allergic reaction leading to a systemic response. Patients are often unaware of such allergies, making allergy testing a recommendation, although not routinely done pre-operatively, with most testing done on a case-by-case basis via patient and surgeon discretion [10]. The standard diagnosing tests for metal sensitivity include cutaneous patch tests and in vitro tests such as the lymphocyte stimulation test (LST), to list a few. The haptens released by nickel ions bind to skin cells, and the cells will produce cytokines, notably IL-1β and TNF-α. The release of cytokines leads to activating antigen-presenting cells such as macrophages which are part of the innate immunity. The activated antigen-presenting cell migrates to the lymph nodes, activating the adaptive immunity, notably the CD4+ T lymphocytes. The CD4+ T lymphocytes, in turn, will activate the CD8+ T lymphocytes and B cells, leading to their sensitization. Reexposure to the hapten again, mediated by the T-cell cellular response, will activate the sensitized cells, causing symptoms of delayed hypersensitivity with reexposure [11].

Systemic nickel allergy syndrome (SNAS) could be a possible causality in this case, as the patient had some neurological and cutaneous effects [12]. Nevertheless, there was a lack of explicit gastrointestinal or respiratory symptoms. The patient’s presenting symptoms did not change with different diets or activities, lending credence to the surgical staples as the possible cause of the symptoms. Removal of the offending agent, as in this patient, is the most effective management highlighted in this case.

## Conclusions

Allergic reactions to metal alloys are a possible adverse outcome during surgical procedures, leading to immediate or delayed hypersensitivity reactions. Increased awareness of medical providers of the potential of metal hypersensitivity can prevent patients from undergoing unnecessary exposure to metal surgical clips. Implementing standard questionnaires regarding metal sensitivities during pre-operative evaluation and additional screening measures such as Metal LTT or patch test can prevent exposure. Surgical suturing is a viable substitution for surgical clips. There have been advances in surgical staples using metal on metal by-products with different coatings or alloys devised to limit metal exposure.
